# Rocket‐shape crossing technique: A combination of lead extraction and modified venoplasty for device upgrade with venous occlusion

**DOI:** 10.1002/joa3.12875

**Published:** 2023-05-24

**Authors:** Junji Morita, Yusuke Kondo, Takuya Haraguchi, Takayuki Kitai, Tsutomu Fujita

**Affiliations:** ^1^ Department of Cardiovascular Medicine Sapporo Heart Center, Sapporo Cardiovascular Clinic Sapporo Japan; ^2^ Department of Cardiovascular Medicine Chiba University Graduate School of Medicine Chiba Japan

**Keywords:** lead extraction, venoplasty, venous occlusion

## Abstract

This case discusses an upgrade method to cardiac resynchronization therapy defibrillator for a 54 year old man with superior vena cava occlusion. Right ventricular lead extraction with modified venoplasty, Rocket shape Crossing Technique (RCT), was performed. In RCT the integration of the inflated balloon, halfway inside the laser sheath, and the laser sheath are advanced through the occlusion like a rocket shape crossing.

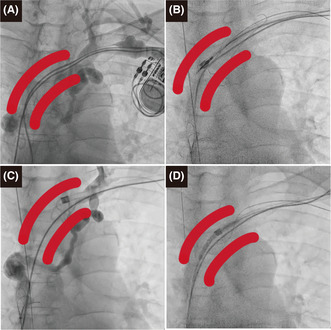

Abu‐El‐Haija et al. evaluated 212 consecutive patients for the presence of venous occlusion and found that it occurred in 26% of the patients with cardiovascular implantable devices during follow‐up.^1^ Interestingly, the use of lead extraction has been described in the Heart Rhythm Society expert consensus statement for device upgrades in patients with venous occlusions.^2^ This statement revealed that some cases require venoplasty for residual occlusion if leads are extracted successfully. Moreover, new leads cannot pass because recoil usually occurs following venoplasty alone.^3^ In this case, we used a modified venoplasty technique that we named the rocket‐shape crossing technique (RCT).

A 54‐year‐old male patient with a history of dilated cardiomyopathy underwent dual‐chamber pacemaker implantation via the left subclavian vein for a complete heart block at the age of 46 years. He presented with progressive exertional dyspnea and a left ventricular (LV) ejection fraction of 20%. The patient's pacemaker data demonstrated 100% right ventricular (RV) pacing, and he was referred to our institution for an upgrade to a cardiac resynchronization therapy defibrillator. Preoperative venography showed occlusion of the superior vena cava and innominate vein (Figure 1A). Lead extraction was performed to provide a channel through which new leads could be implanted. The RV lead was extracted using a 14‐Fr excimer laser sheath (GlideLight™; Spectranetics Corporation) (Figure 1B). After extracting the RV lead, the laser sheath was within the venous occlusion, as shown by contrast through the tip of the laser sheath (Figure 1C). Venoplasty was required to pass new leads, and we attempted to pass through the occlusion using the guidewire. We found that it was possible to pass the guidewire by following the path created after removing the RV lead. Initially, we attempted to pass through the subclavian vein; however, this was unsuccessful. Therefore, we approached from the femoral vein and inserted the guiding sheath and guidewire. The guidewire was successfully passed through the occlusion and advanced into the excimer laser sheath in the subclavian vein, ultimately exiting the body. We initially inflated a 4 mm × 40 mm balloon (Mustang™; Boston Scientific) to dilate the occlusion; however, the laser sheath could not pass through. Therefore, we advanced the balloon into the excimer laser sheath for half its length. The balloon was inflated to dilate the occlusion, while the balloon and laser sheath were successfully integrated to round the edge of the laser sheath and pass through the occlusion safely, resembling a “rocket‐shape crossing,” without laser ablation (Figure 1D) (Supplementary video 1). After the “rocket‐shape crossing,” new sheaths were advanced easily into the right atrium, and the new implantable cardioverter‐defibrillator and LV leads were implanted.

**FIGURE 1:**
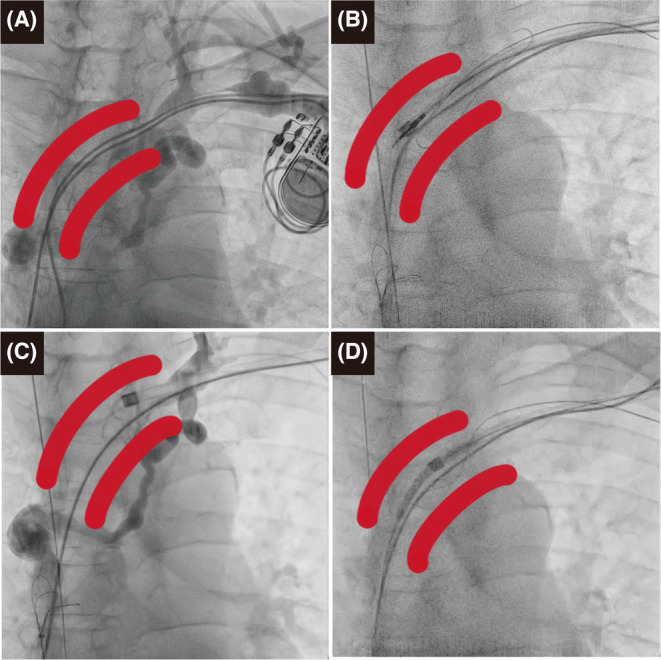
(A) Venography demonstrating occlusion of the superior vena cava and innominate vein. (B) Successful lead extraction. (C) Laser sheath within the occlusion of the innominate vein. (D) Passing through the occlusion using the “rocket‐shape crossing” technique.

To our knowledge, this is the first case where the modified venoplasty technique was used. The RCT is a procedure where the inflated balloon is integrated halfway into the laser sheath, and the laser sheath is passed through the occlusion, similar to a “rocket‐shape crossing” without laser ablation. An advantage of the RCT is that the gap between the balloon and the laser sheath becomes smaller, and the edge is rounded by the inflated balloon. Therefore, the balloon size should be equivalent to, rather than exceed, the laser sheath's inner diameter to prevent sheath injury. Additionally, the laser sheath is more likely to pass through when the diameter difference between the sheath and the balloon is minimized. In this case, we selected a short length and opted for a 4 mm × 40 mm balloon for the laser sheath (14‐Fr, 50 cm), ensuring that the balloon size was equivalent to rather than exceeding the sheath's inner diameter. Furthermore, a smooth transition improves the crossability of the occlusion and decreases the risk of vein injury and scratching of the intramural thrombus. Moreover, even when laser ablation is not applied to remove the leads, this technique remains utilizable with general mechanical sheaths.

In summary, we described the utility of modified venoplasty during device upgrades in patients with venous occlusions. Further studies are warranted to elucidate the associated benefits and risks of modified venoplasty.

## FUNDING INFORMATION

This research did not receive any specific grant from funding agencies in the public, commercial, or not‐for‐profit sectors.

## CONFLICT OF INTEREST STATEMENT

Dr. Kondo received lecture fees from Daiichi‐Sankyo, Bayer, Abbott Medical Japan, Biotronik Japan, Boston Scientific, and Japan Lifeline, and research funds from Daiichi‐Sankyo. The other authors have no conflicts of interest to declare.

## ETHICS STATEMENT

This case report was approved by the institutional review board and followed the principles of the Declaration of Helsinki.

## PATIENT CONSENT STATEMENT

The patient provided written informed consent.

## Supporting information


Supplementary video 1
